# Level of satisfaction and sexual and reproductive health needs of deaf persons in Ghana: a sequential explanatory mixed method study

**DOI:** 10.1186/s12913-022-08515-z

**Published:** 2022-09-12

**Authors:** Wisdom Kwadwo Mprah, Maxwell Peprah Opoku, Juventus Duorinaah, William Nketsia

**Affiliations:** 1 Centre for Disability and Rehabilitation Studies, Department of Health Promotion and Disability Studies, Kwame Nkrumah University of Science and Technology, Kumasi, Ghana; 2 Special Education Department, United Arab Emirates University, P. O. Box 15551, Al-Ain, United Arab Emirates; 3 Ghana National Association of the Deaf, Accra, Ghana; 4 School of Education, Western Sydney University, Sydney, Australia

**Keywords:** Deaf persons, Sexual and reproductive health, The hierarchy of needs, Culture, Education, Ghana

## Abstract

**Background:**

The intersection between deafness and culture in sub-Saharan African contexts such as Ghana has culminated in restricted access to sexual and reproductive health (SRH) services. While some attention has been given to the barriers faced by deaf persons in accessing SRH services, discussion of their needs and satisfaction with SRH services is at an embryonic stage. This lends support to the use of sequential mixed-method study design to assess the level of satisfaction and SRH needs of deaf persons.

**Methods:**

This study was guided by explanatory sequential mixed-method study design. Thus, a two-phase data collection approach was adopted. In Phase I, a 32-item questionnaire with 16 items each for satisfaction regarding SRH services and SRH needs, was used for data collection from 288 deaf persons recruited from 3 of the 16 regions in Ghana. The data were subjected to the following computations: means, *t*-tests, analysis of variance, correlations, and multiple regression. In Phase II, a semi-structured interview guide was used to collect data from 60 participants who were drawn from the earlier pool. The interviews were subjected to thematic analysis.

**Results:**

The results showed of correlation and multiple analyses showed a small relationship and significant contribution of needs in the variance of satisfaction. Also**,** there was a convergence between both the qualitative and quantitative data as participants confirmed the lack of consideration given to the needs of deaf persons regarding SRH service provisions.

**Conclusion:**

Deaf persons who took part in this study were unsatisfied with SRH services due to barriers such as sign language interpreters and inaccessible information. Consequently, they expressed the need for preferred mode of communication and expedition of awareness creation on SRH. The study findings warrant the need for policymakers to inculcate the needs of deaf person in SRH services to improve access and thus, enhance satisfaction. For instance, recommendations such as the training of health professionals in the use of sign language could be considered in future SRH policy and other implications, are discussed.

## Background

Sexual and Reproductive Health (SRH) refers to one’s state of physical, emotional, mental, and social well-being on matters relating to their reproductive system. Access to SRH services has been acknowledged widely as a human right [1–7]. The International Conference on Population and Development in Cairo in 1994 drew the world’s attention to the need to uphold and protect this right [1]. It asserts that individuals should be free to make decisions including those regarding a partner, consent to safe sex, and choosing the number and spacing of children [8]. In view of this, the realization of SRH as a human right ensures that women are safe from sexual exploitation, eliminates preventable maternal deaths, and ensures that reproductive health services such as contraceptive services, family planning counseling, and safe abortion services are available to all [6–8]. While some countries have taken steps to optimize the prioritization of SRH [9–13], other countries are being urged to move from political rhetoric and take concrete steps regarding enabling services for all [9, 13]. However, there are still gaps in service provisions for minority groups such as deaf persons at high risk of exclusion from SRH policies [11, 12].

Deafness refers to partial or total hearing loss that interferes with the day-to-day living experience of an individual [14–16]. According to the 2021 Population and Housing Census, 8% of the estimated 30 million people are living with a form of disability [17]. It has been reported 22% of the 2million disability population are living with hearing impairments with females constituting 59% compared to 41% for males [17]. The inability of deaf persons to communicate or be receptive to information from others has severe impacts on their ability to access essential services such as education, employment, healthcare, and transportation [18–25]. In healthcare, a small body of literature has reported the inability of both deaf males and females to access SRH services [20–22, 26–28]. More so, it has been reported that the major needs of deaf persons in effort towards accessing SRH services is communication need [21, 22]. However, it is apparent that communication need is yet to be met which puts deaf persons at risk of death, teenage pregnancies, and adoption of unsafe health practices [21, 22, 28–32]. The main reason accounting for the unmet need of deaf persons is cultural understanding of deafness. In the sub-Saharan Africa context, conditions such as deafness intersect with the culture of the society [18–26]. The birth of a deaf child is perceived as an orchestration of supernatural forces. As a result of this notion, deaf children are highly discriminated against and subjugated in society [23, 24]. This cultural understanding has an effect on the child’s socialization and subsequent inclusion in the larger society. For instance, in the development of policies on essential services, their needs or services are less likely to be considered [18]. Also, sign language is not recognized as a form of language, which makes it hard for them to be understood by others in society [22]. Professionals such as healthcare workers and teachers do not have proficiency in sign language to communicate with them [20–22].

Currently, as part of the effort regarding achieving a safe world for all, the United Nations, through Sustainable Development Goals (Goal 3), has urged countries to develop a robust system that would enable all persons to have access to essential services such as healthcare [33–35]. In Ghana, there has been policy formulations such as National Reproductive Health and Service Policy and Standards, National HIV and AIDS, STI Policy, Ghana National Condom and Lubricant Programming Strategy, the National Gender and Children Policy, the Ghana National Reproductive Health Commodity Security Strategy 2011–2016 and Adolescent Reproductive Health Policy [36], to promote the SRH services to the general population. Although these policies have been hailed as pivotal in effort towards improving access to SRH, there is still gaps in access to the general population [28–32]. For instance, teenage pregnancy rate is estimated at 14%; contraceptive usage is 20%; and maternal deaths is 310 per every 100,000 births [36]. These disturbing figures raise critical issues in terms of the plight of deaf persons which is not captured in national report. Indeed, these policies have been critiqued for not directly addressing or including the SRH needs of deaf persons [20–22].

In low- or middle-income contexts, little attention has been given to the experiences of deaf persons [20–22, 26, 37] and persons with other disability conditions [37–40] in accessing SRH services. In a Nepalese mixed-method study, 384 persons with disability of reproductive age to understand factors affecting the utilization of SRH services [38]. The finding showed inaccessible health facilities and marital status impacted access to services. Follow-up interviews showed that illiteracy among the disability groups, lack of family support, and other factors negatively impacted SRH service access. Similar challenges have been reported in Ghana when it comes to promoting access to SRH to persons with disabilities [20–22, 26, 39–44]. For example, Mprah [20] used a qualitative method to explore the experiences of deaf persons with SRH services in Ghana. Factors such as illiteracy, communication barriers, lack of privacy, and negative attitudes of health workers regarding deaf persons were notable barriers to SRH services. Other studies have reported challenges to SRH to general disability groups [40, 41] such as those with visual and cognitive disabilities [42, 43]. Among the general population, studies have reported challenges to SRH services [36, 45–49]. Since individuals with disabilities are a heterogeneous group [15, 16, 18], there is a need to study them individually. Although studies on access to SRH to persons with disabilities have received some attention, research on the level of satisfaction and SRH needs is very rare.

In this study, while needs were operationalized as lack of SRH services, which are essential to enabling deaf persons to enjoy appropriate reproductive health, satisfaction was conceptualized as meeting one’s needs. We hypothesized that once the SRH needs of deaf persons are met, satisfaction regarding SRH services will be high. The hypothesis is supported by Abraham Maslow’s hierarchy of needs, which states that humans are motivated by needs [50–52]. When one need is satisfied, individuals will strive to achieve the next need on the hierarchy. The needs are ranked in this order: physiological, safety, love/belonging, esteem, and self-actualization [51]. Although the deaf persons are often underserved regarding basic needs such as food [18], we deemed SRH a safety need that is required to enable them to take control of their reproductive system. The availability of SRH services would enable them to have a sense of security and be confident in the health systems that safeguard their SRH. Thus, we hypothesized that when needed SRH services are provided, satisfaction regarding SRH will also increase. To test this hypothesis, the study was guided by the following research questions:

- RQ1. What is the association between background variables, SRH needs, and satisfaction regarding SRH services among deaf persons in Ghana?
- RQ2. Will the SRH needs of deaf persons directly predict satisfaction with SRH services among deaf persons in Ghana?
- RQ3. How do deaf persons perceive their SRH needs and their level of satisfaction regarding SRH services provided to them?

## Methods

The study was guide by a sequential explanatory mixed-methods design. Data were collected in two phases to enable an in-depth insight into a given problem or phenomena [53–55]. Thus, after the first quantitative phase, follow-up interviews were conducted selected participants who took part in the initial phase. This was intended to give participants the chance to add their voice or clarify key trends in the initial phase [53–55].

The study was conducted in two phases. In phase one, data were collected from participants (deaf persons) who were recruited from various districts. In the second phase of the study, interviews were conducted with selected participants who took part in phase one. This was to enable participants to clarify and provide an in-depth explanation of the key trends that emerged in phase one. The sections below present participants’ information, data collection instruments, and procedures as well as data analysis techniques.

### Participants selection

Participants in this study were selected in collaboration with the Ghana National Association of the Deaf (GNAD), an association that seeks to promote the welfare of deaf persons in Ghana. Prior to the selection, a meeting was held with GNAD, who recommended the recruitment of six districts in three regions (two from each). GNAD believed that the regions suggested for the study represent three main categorizations in Ghana: north (Northern region), middle belt (Ahafo region), and southern sectors (Greater Accra region). The selection of these regions helps recruit participants which may reflect the national population and thus, provide insight into deaf persons in Ghana.

The two districts from each region as well as the participants recruited were chosen at random. The list of the districts in the regions were placed before the study research team who random chose the first and last districts on the list. The inclusion criteria which guided the recruitment were as follows: a) participants are members of GNAD; b) lives in the designated areas; c) at least 18 years; and d) capacity to participate in this study. All members of GNAD who met the inclusion criteria were sent invitation and those who agreed to participant in this study visited the organization’s office on weekends to complete the questionnaire. This gave every member present a chance to be part of the study.

### Instrument

Phase I: A two-part questionnaire was used for data collection. Part one collected participants’ background information, which includes gender, age, educational qualification, marital status, and religion. This background information was collected based on a review of existing literature (e.g., [27–30, 40–49]) and advice from GNAD regarding the collection of information from deaf persons.

The tool for data collection was made up of two sections. The first part was items on the needs of deaf persons. This section was made up of 16 items anchored on a binary scale (1 = important; 2 = not important). Some of the items on the scale were “I want easy access to education on sexual and reproductive health,” “Sign language interpreters at sexual and reproductive health centers/programs to interpreter sexual and reproductive health information for me (and other deaf people),” and “Sexual and reproductive health messages should be presented in simple and accessible formats such as dramas, videos, and pictures.” All the items were positively worded with a mean score of at most 1.5 interpreted as more needs. The 16-items on the scale were added to generate overall scores for needs [56].

The second part was items on satisfaction regarding SRH services. The section also contained 16 items using a binary scale (1 = satisfied; 2 = not satisfied). The following are some examples of items: “How satisfied are you with access to sexual and reproductive health education?,” “How satisfied are you with the current form in which information on sexual and reproductive health is presented to you?,” and “How satisfied are you with the current respect you receive from health workers?” During analysis, the 16-items on the scale were added to generate overall scores for satisfactions [56].

Delphi technique (which is a content review of the questionnaire by experts) [57] involving three academics and three members of the deaf community in Ghana and the United States was employed for analysis. The suggestions from the experts were incorporated into the instrument prior to data collection. One key suggestion was rewording some questions, reducing the anchors in the questionnaire to two, and deleting some demographics (e.g., employment status).

The questionnaire was then piloted among 10 participants from different regions. The piloting yielded the following reliability scores: SRH needs (.70) and satisfaction regarding SRH (.88). In this study, the reliability scores as computed using the Cronbach Alpha were as follows: SRH needs (.91) and satisfaction regarding SRH programs (.89).

Phase II**:** Based on the emerging issues in phase one of the study [53–55 ], an interview guide was developed to cover participants’ perception of needs and satisfaction regarding SRH services. Some of the questions asked included: “In the first phase, deaf persons appear to have more SRH needs. What do you think about this?,” “Deaf persons appear to be unsatisfied with SRH services provided to you. What is your opinion about this?” and “In the first phase, we noted that as needs regarding SRH are met, deaf persons would be more satisfied with SRH services. Do you agree with this?”

### Data collection

The research team indicated to GNAD that they were interested in recruiting heterogeneous participants. Upon consensus with GNAD regarding study areas and heterogeneity of participants, the organization (GNAD) requested their regional and districts representatives, who invited their members to be part of this study. The members of GNAD has a shared social media platform, WhatsApp Groups, where invitations were sent out to all members. Subsequently, a list of potential participants who agreed to participant in this study was given to the research team, who invited all prospective participants via texts and video WhatsApp messages. The inclusion of video messages provided a sign language version of the information to participants for understanding or to participants who might not be able to read.

Phase I: The data was collected from June 2016 to December 2018. Two research assistants were recruited to provide support to participants in completing the questionnaire. The research assistants were trained graduate students who are proficient in Ghana Sign Language and English. Information sheets explaining the study were sent to participants via texts and video (WhatsApp) or email. The questionnaire was completed at designated places (GNAD district office) on weekends. Prior to this, participants were encouraged to visit the GNAD office to complete the questionnaire at their own convenient time within a given period. All participants completed a hard copy of the questionnaire with or without assistance. The participants were provided with breakfast and lunch and some received cash reimbursements (equivalent of US$ 10) after completing the survey to cover their transportation cost to the data collection centers.

Phase II: The participants for this second phase were drawn from the pool who took part in the first phase. The research team held a meeting with GNAD to discuss recruitment strategy and procedure. After reaching a consensus to select 10 participants from each district, random invitations were sent to participants until the required number was reached. Interview dates and times were scheduled with the consent of participants. It was agreed that there would be one focus group discussion made up of seven people while the remaining three were engaged in one-on-one interviews. The interviews were videotaped with permission from the participants.

### Data analysis

Phase one: Prior to data analysis using SPSS (version 28), the completed questionnaire was manually entered into Excel and cleaned. Since the study had a large sample size, we did not anticipate violating the assumption of normally, and thus the data was appropriate for the parametric test. To answer research question one and understand the predictors of SRH service satisfaction, the quantitative data was analyzed as follows: computation of mean scores, *t*-tests and analysis of variances (ANOVA), and linear regression.

The sum of the items was used for analysis. For instance, the 16-items each on the needs and satisfaction scales were summed before analyzing the data. The mean scores were computed to understand participants’ needs and satisfaction regarding SRH service. To answer research question one, *t*-tests (for two-level demographics) and ANOVA (for at least three level demographics) were computed to understand [56] the difference between participants on SRH needs and satisfaction. For the *t*-test, the assumption of homogeneity of variance was checked using Levene’s test, which showed no violation for all the computations [56]. The weight of the results was assessed using Cohen’s *d,* which was interpreted as follows: small (.10 to .29), moderate (.30 to .49), and large (.50 to 1.0). On ANOVA, in the event of a violation of homogeneity of variance, the results of Welch statistics were reported [56]. Here the effect size was assessed using partial eta squared, interpreted similarly to the Cohen’s *d*.

To answer research question 2, the predictors of SRH were computed using linear regression. However, before doing that, the relationship between the measures was assessed using Pearson Moment Correlations whose results were interpreted as follows: small (.10 to .29), moderate (.30 to .49), and large (.50 to 1.0) [58]. Following this, hierarchical multiple regression was computed to understand whether needs will predict satisfaction regarding SRH services. While needs were regressed directly on satisfaction regarding SRH services in step 1, other demographics were added to needs to assess its impact on satisfaction regarding SRH services. The following assumptions were assessed to ensure they were not violated: linearity, multicollinearity, and homoscedasticity [58].

Phase two: The videotaped data were saved on a computer for transcription by research assistants. Afterward, the interview transcripts were sent to some of the participants who provided feedback and suggested iteration where necessary [59]. More so, discussion of the salient points from the interviews were conducted with participants through video WhatsApp calls. Participants were satisfied with the information and agreed to its use in the reporting.

At this stage, the data were subjected to thematic analysis [60]. It is worth stating that the framework for the development of the interview guide was used as an a priori theme to guide the analysis process. The data analysis involved the following stages: reading and coding, sorting and mapping, categorization, thematizing, and writing results. In stage one, authors two, three, and four read the transcribed data several times and shared major emerging points with the research team.

The next stage was coding the transcribed data. After coding one focus group discussion and one interview, the authors met to discuss descriptors used for the coding. Where disagreement arose between authors, a consensus was reached before continuing the coding of the remaining data. The second stage involved sorting and mapping using emerging sub-themes. This enabled the authors to map common ideas and identify areas of disagreement between the participants. The next stage was categorization where the authors grouped the sub-themes that were subsequently charted under the priori themes. Selected quotes explaining the categories were transferred onto a new file. The results section was written by author two and shared with all the authors for their reading, changes, and approval.

### Ethical considerations

The Committee for Human Research and Publication at Kwame Nkrumah University of Science and Technology reviewed and approved the study protocols (CHRPE/AP/375/16). Further approvals for the study were given by GNAD and Ghana Health Service before implementation. The participants were provided with consent information that clearly stated their right to withdraw from the study at any time without consequences and assurance of confidentiality and anonymity. Also, the risk involved in the conduct of the study and potential benefits towards health reforms were explained to participants before completing the questionnaire. All participants provided either oral or written consent before taking part in the study.

## Results

Phase I: Out of 360 questionnaires distributed to the participants, 288 were returned, representing an 80% return rate. In terms of gender, 75% were female, while 25% were male. The majority of participants (59%) were between 17 and 25 years of age compared to 15% who were at least 36 years of age. With respect to education, 50% of the study participants had basic qualifications, whereas 11% indicated they had tertiary qualifications. Also, 71% indicated that they were married, while 29% were single. Christians made up 68% of participants, while 32% specified they were Muslims (Table 1).

**Table 1:** Summary of demographic characteristics of study participants

***N*** = 288	Sample	%
**Gender**		
Male	72	25%
Female	216	75%
**Age**		
17–25 years	171	59%
26–35 years	74	26%
36 years and above	43	15%
**Educational qualification**		
Basic^a^	144	50%
Secondary	113	39%
Tertiary qualification	31	11%
**Marital Status (*n*** **= 2)**		
Single	82	28%
Married	206	72%
**Religion (*n*** **= 2)**		
Christianity	192	67%
Muslim	90	33%

^a^Basic means either completed or drop out of primary and junior high school

### RQ1: association between demographics, needs, and satisfaction

The computation of the mean scores showed the following results: needs (*M* = 1.35; *SD* = .38) and satisfaction (*M* = 1.79; *SD* = .33). This suggests a level of dissatisfaction with the SRH services provided by health professionals.

The relationship between participants’ profiles, needs, and satisfaction regarding SRH services were measured using *t-*tests (e.g., gender) and analysis of variance (ANOVA; e.g., age). While the *t*-test showed no relationship between the two-level demographics and the measures, the results of the ANOVA showed an association between age and SRH needs only: *F* (2, 285) = 4.02, *p* = .02 with very small effect size, partial eta squared = .03. Post-hoc comparison using Tukey HSD test showed that the younger the participant, the more needs expressed compared to much older deaf participants (see Table 2 for details).

**Table 2:** Association between demographics, needs and satisfaction

***N*** = 288	Needs	Satisfaction
**Gender**		
Male	1.39 (.42)	1.79 (.38)
Female	1.34 (.36)	1.78 (.32)
*t*	1.062	.07
Cohen’s *d*	.15	.01
**Age**		
17–25 years	1.37 (.32)^a,b^	1.77 (.30)
26–35 years	1.26 (.37)^b^	1.78 (.33)
36 years and above	1.46 (.56)^a,c^	1.85 (.46)
*F*	3.06#*	.89#
Partial eta squared	.03	.01
**Educational qualification**		
Basic*	1.34 (.38)	1.80 (.32)
Secondary	1.34 (.33)	1.75 (.32)
Tertiary qualification	1.48 (.53)	1.86 (.44)
*F*	1.96	1.78
Partial eta squared	.01	.01
**Marital Status (*n*** **= 2)**		
Single	1.35 (.40)	1.78 (.35)
Married	1.35 (.37)	1.79 (.33)
*t*	.02	−.12
Cohen’s *d*	.002	.02
**Religion (*n*** **= 2)**		
Christianity	1.34 (.40)	1.81 (.34)
Muslim	1.37 (.32)	1.75 (.28)
*t*	−.63	1.39
Cohen’s *d*	.08	.18

*p < .05; **p < .01; superscripts ^(abc)^ = significant difference between participants; #violation of the assumption of homogeneity for ANOVA and reporting of Welch Statistics; *Basic means either completed or drop out of primary and junior high school

### RQ2: predictors of needs and satisfaction

Prior to conducting hierarchical multiple regression, Pearson moment correlation was conducted to understand the relationship between needs and satisfaction regarding sexual and reproductive health services. The results showed a small relationship between needs and satisfaction (*r* = .30, *p* = .001).

Afterward, hierarchical multiple regression was computed to determine whether the needs of deaf persons would predict satisfaction while controlling for the influence of other demographics (Table 3). In the first step, needs (beta = .28) made a significant contribution of 8% in the variance in satisfaction, *F* (1, 279) = 22.84, *p* = .001. In step 2, five demographic factors were added to the model: gender, age, education, marital status, and religion. The demographic variables made an additional contribution of 2% in the variance in satisfaction, *F* (5, 274) = 5.12, *p* = .001. While only age made a significant contribution in the variance in satisfaction (beta = .14, *p* = .05), needs regarding SRH (beta = .28) once again made the greater contribution to the variance in satisfaction.

**Table 3:** Predictors of satisfaction

Category	B	S. E	Beta	***t***	***p***
**Step 1**					
Needs	.24	.05	.28	4.78	.001**
**Step 2**					
Needs	.24	.05	.28	4.89	.001**
Gender	.49	.68	.04	.71	.48
Age	.87	.49	.14	2.01	.05*
Education	−.56	.48	−.07	− 1.17	.25
Marital status	.41	.77	.04	.53	.60
Religion	−1.17	.64	−.11	−1.83	.07

*p < .05; **p < .01

Phase II: In all, 60 participants comprised of 18 males and 42 females took part in the second phase. Six focus group discussions (*n* = 42) and 18 face-to-face interviews were conducted. Eight (Table 4).

**Table 4:** Demographic characteristics of study participants

Categories	Sample (***N*** = 60)
**Mode of participation**	
Focus Group	42
One-on-one interview	18
**Gender**	
Male	18
Female	42
**Age**	
17–25 years	33
26–35 years	10
36–45 years	11
46 years and above	6
**Religion**	
Christian	34
Muslims	22
Other	4
**Educational level**	
Basic level*	31
High school level	16
Tertiary qualification	13
**Employment**	
None	26
Student	5
Self-employed	19
Public service	10
**Marital Status**	
Single	26
Married	29
Divorced	5

*Basic means either completed or drop out of primary and junior high school

### RQ3: result

Participants were asked to share their perspectives on key findings emerging from the first phase. Indeed, participants enumerated a number of challenges serving as a barrier to their access to SRH information. They felt dissatisfied with the services received and expressed the need for improved services.

### Level of satisfaction

Almost all the participants reported low satisfaction regarding SRH services. They indicated barriers encountered in accessing SRH services. Some participants said they were satisfied with the services received, but a large number of them were not satisfied. A few participants in both the focus groups and interviews indicated that deaf people were aware of the problems associated with unprotected sex, unwanted pregnancies, and unsafe abortion and even discussed these with their trusted friends. Those who were not satisfied said they did not benefit from the services they received because of a lack of sign language interpreters. They explained that without interpreters, they would “never understand or benefit from services offered by health workers and so they got low satisfaction from the services being offered” (Female, Interview Participant 2, District E). Agreeing with the above assertion, a focus group participant said that “we are never satisfied because we often make guesses to understand the nurses in instances where there are no sign language interpreters” (Female, Focus Group Participant 5, District D). Two participants elaborated:

*Lack of sign language interpreters in such places [SRH centres] is the number one hindrance that always prevented deaf people from getting information and services on SRH issues from these sources that claim to be focusing on the SRH needs of people. They do not offer services that satisfy deaf people (Female, Focus Group Participant 5, District A).*

*We are not satisfied because many information and services the hearing people get are better than what the deaf people receive due to communication barriers, and so, I want them [SRH providers] to come and train us in order for us to give more information and services and also to enable us to support other deaf people elsewhere (Male, Focus Group Participant 2, District F).*

Many participants said they distrust the information they got from the various sources: “Most of us do not trust them [service providers] because we do not understand what they say on most of the health-related issues” (Female, Interview Participant 2, District D). Another female participant also said: “I do not trust them [services on SRH issues] because I think they may not explain things well for us to understand” (Female, Interview Participant 3, District C). Participant in the focus groups similarly explained that they did not trust their sources of information.

*I do not trust 100% of the information and services on SRH issues I have because I do not fully understand the issues and, also, I think there are more things to learn about SRH issues. I do trust the source because the facilitators cannot share some of their experience because of communication barriers (Female, Focus Group Participant 7, District A).*

Other participants also recounted that their low satisfaction could be attributed to a limited level of education. In their view, most deaf women are uneducated and, as such, cannot read to understand the effects of self-medication or other accessible written information.

*Some of us never went to school before and as such could not sign and write well. Therefore, it becomes a problem when we are inquiring for information and services on any of the SRH services from health workers because of lack of sign language interpreters (Male, Interview Participant 2, District F).*

### SRH needs of deaf persons

There were mixed reactions from participants on the age group that is more likely to have more SRH needs. While most participants agreed that the young people would have more needs, some said that older young persons have more SRH needs. According to some participants, young deaf persons are more likely to be educated and more aware of their SRH needs. However, some participants said that deaf persons have the least education, and, in view of this, it is likely that young persons would not have much information of SRH. In terms of older persons, some participants said that they have experience that might have helped them develop an understanding of SRH. Overall, there was a consensus among participants that both young and older deaf persons are more likely to have their SRH needs unmet. Almost all participants indicated the need for policymakers to incorporate their needs in national SRH development and planning. Consequently, improved SRH services would enable deaf persons to enjoy their right to SRH. Indeed, most participants agreed that they would be more satisfied with SRH services once their needs are incorporated in policy reforms. Two main themes about needs emerged: using the preferred mode of disseminating information and awareness creation on SRH issues.

### Preferred modes of disseminating information

Responses from the participants indicated that their preferred sources of SRH information were teachers, healthcare centers, and the media. But it appears hospitals would have been the preferred sources if they were deaf-friendly. The following quotes support this viewpoint.

*Health workers and other reproductive health facilitators and teachers are the preferred sources of information for deaf people. This is because they are experienced [on SRH issues] and do share important issues with us, so we want them to provide information on SRH for us but they should use interpreters to facilitate understanding (Female, Focus Group Participant 5, District E).*

*There is no organization that can provide information and services on SRH for the deaf people aside from hospitals. This is because hospitals are the only places where we know we can get good information and services on any of the health issues that we may want to know more about (Male, Focus Group Participant 1, District B).*

On the preferred means of disseminating information from these sources, participants mentioned using sign language interpreters, videos, drama, storytelling, and pictures. In addition, health professionals should be encouraged to learn the sign so that they can communicate directly with deaf people. Using these modes of communication would improve access to quality services for deaf people. One participant recounted as follows:

*Direct communication with health workers will be more useful because it will make deaf people more conformable. They should also try to train more interpreters and get more pictures with storylines on SRH issues with sign language symbols. Doctors and nurses should make the effort to learn the sign language so that they can communicate effectively with the deaf people on SRH issues. (Female, Focus Group Participant 6, District F).*

### Awareness creation on SRH

The provision of education on SRH issues was suggested by participants as the best way to increase knowledge on SRH issues. Participants were unanimous that education would enable deaf people to know more about their rights to health, which will, in turn, encourage them to ask more questions and hold professionals accountable. A focus group participant commented:

*There should be education and materials on SRH issues, and also Ghana Health Service should visit church camps, churches, schools and communities to give information to deaf people on SRH issues. Deaf people should also be educated on their right to information and reproductive health issues. With education, I believe they will know their rights and be motivated to seek for information and services on SRH issues. (Male, Focus Group Participant 5, District C).*

Access can also be improved through the use of the appropriate formats: “When conducting education on sexual health, bright images, dramas, and banners should be used because it will make them interested to seek for more information on their SRH” (Male, Interview Participant 2, District). A female interview participant also suggested that “Captions for videos should be used and there should be continuous sensitization on matters concerning reproductive health” (Female, Interview Participant 1, District C).

## Discussion

In this study, attempts were made to develop in-depth insight into the satisfaction regarding SRH services and the SRH needs of deaf persons. The study findings support the hypothesized relationship between SRH needs and satisfaction of deaf persons regarding services. The results of the correlation, regression, and follow-up interviews showed the centrality of needs in achieving satisfaction with SRH services. In the conception of the theory of needs, Maslow reiterates the intricate relationship between needs and satisfaction [50, 51]. The finding was expected in the sense that deaf persons struggles with basic needs, which suggests that policymakers have done little to expand safety services in areas such as SRH. Due to the social construction of deafness as a consequence of sin [22–24], deaf persons are usually left out of major development initiatives and programs. It is clear that more needs to be done on improving SRH services to meet the expectations of deaf persons who are usually left of major policy instruments. There is the need for minority-friendly SRH policies, targeting individuals like deaf persons, to guarantee meaningful access to services for all.

The quantitative and qualitative data showed convergence between participants on satisfaction regarding SRH services. In particular, satisfaction regarding SRH services was found to be low, claim participant confirmed in the second phase of the study. This finding is slightly consistent with other studies, which reported the inability of health systems to meet the needs of individuals with disabilities, including deaf persons [27, 28, 39, 40]. This finding is expected in an environment where disability has an intersection with the local culture [25, 43]. According to Maslow’s theory of needs, once individuals are satisfied with one need, they can move on to pursue higher needs [50, 51]. The dissatisfaction among participants regarding SRH services could mean that they would be hesitant to pursue other higher needs such as love or self-esteem. The findings underscore the need for policymakers to integrate the needs of deaf persons in future attempts to revamp systems to enable accessible SRH services to all in the provision of services.

The results of this study showed participants’ expression of more SRH needs. It is apparent that the more needs of participants, the more current service provisions are inadequate to address the diverse needs of deaf persons. It is useful to state here that some studies have reported the lack of consideration given to the needs of deaf persons in the development or provision of essential services [22, 26, 27, 40]. According to Maslow, every person is motivated by needs [50]. Thus, in the event the needs of deaf persons are included in SRH service provision, they would be able to appreciate the services and facilitate utilization of services. SRH services are broad [7–9], which means that deliberate effort ought to be made to integrate the needs of deaf persons in each of service. This finding probably calls for health system reformation through deliberate integration of the needs of deaf persons in the policy document on SRH.

The participants suggested needs to be included in future SRH policies. For instance, they indicated a preferred mode of communication and awareness creation on SRH as vital to meeting their needs. It is important to state here that a large body of literature has documented challenges to SRH service access to include illiteracy, inaccessible information, and lack of education on SRH [7, 11, 61–64]. In the Ghanaian context, deaf persons face major communication hurdles in their attempt to interact with others members of society or receive information on matters relating to them [37, 40]. This is against the backdrop of limited avenues for teaching sign language to members of society [21, 22]. Lack of communication between deaf professionals and health professionals could have consequences on their ability to access useful SRH services or education. This could potential put deaf persons at risk of adopting inappropriate SRH practices. Thus, it would be useful for future SRH policies to consider the communication needs of deaf persons at health facilities and leverage their leadership to extend awareness creation drive to them.

One background variable that was found to impact satisfaction regarding SRH is age. As participants grow, they more likely would be unsatisfied with SRH services—a trend that is unexpected. Although previous study has reported the likelihood of aged persons with disability to utilize SRH services [38], this study has shown that they might not be satisfied with the service they were provided. The expectation is that the older the deaf persons, the more likely they would be to have experience, develop knowledge of SRH, and be able to support their dependents. However, this finding could be explained by twofold reasons. First, there is the likelihood that the young deaf persons may be in a school where they might have learned about SRH. If such a trend is due to education, it means that most of the aged persons may be out of school or have limited access to education. This is pertinent because in Ghana, many deaf adults are mainly uneducated and unaware of basic health needs [21, 22]. The second reason could be attributed to the fact that as deaf persons are growing, their SRH needs grow. These two-sided explanations possibility necessitate the need for tailored awareness programs for both young and older deaf persons.

### Study limitations

In spite of the contribution of this study to research on deaf persons, the findings should not be generalized to the whole population due to its limitation. First, all the participants in the study were members of GNAD; hence, non-members were not given the opportunity to express their views. Deaf persons who are not members of GNAD could have a contrary view to what has been reported in this study. However, it is worthy to state that the research team deemed it appropriate to collaborate with GNAD in conducting this study because it is a recognized body involved in advocacy for and promoting the well-being of deaf people. Additionally, the participants were from diverse backgrounds; therefore, their experiences could reflect other members of society. Second, the study could not ascertain whether participants were only deaf or had an additional disability. Since the research team did not have the capacity or resources to conduct a hearing assessment, it was prudent to depend on a recognized body such as GNAD to assist in identifying potential participants. Third, since GNAD is actively involved in a section of the study and recruitment of participants, there is a potential bias. It is possible a section of participants who could provide certain responses were invited or the executives did not follow the inclusion criteria in choosing participating districts. Nevertheless, inclusion criteria and selection of participants for the interviews were done solely by the research team. It is therefore recommended that future studies use quantitative design to understand background variables that may influence deaf persons’ perceptions regarding access to abortion services. Overall, a major strength of this study was recruiting male and female deaf persons to share their perspectives on this sensitive topic.

## Conclusions and study implications

This study was conducted using Maslow’s [51, 52] hierarchy of needs a to assess the SRH needs and satisfaction of deaf persons. The findings showed a relationship between SRH needs and satisfaction towards SRH thus, confirming our initial hypothesis. Also, there was association between age and satisfaction. In particular, there was age difference between participants on needs and age emerged as significant predictor of satisfaction. In the following up interviews, participants discussed challenges affecting their ability to access SRH services. The participants were unanimously unsatisfied with SRH needs and expressed several needs including communication and need for awareness creation.

The findings of the study have implication for policymaking and practice. For example, the communication needs of a deaf person in health service provisioning is vital, as it has consistently been identified as a gap [20–22, 25, 37]. Health workers usually struggle or do not have proficiency in the use of sign language to communicate with deaf patients [20–22]. It is, therefore, time policymakers consider training health professionals or employing individuals with proficiency in sign language at health facilities. In this way, the communication barriers faced by deaf persons may be overcome. Additionally, policymakers may consider partnering with GNAD to provide accessible SRH education to deaf persons. Here, individuals with proficiency in sign language may be tasked to provide such training services to deaf persons. An effective partnership would enable GNAD to draw the attention of policymakers to other concerns such as financial barriers, which might restrict access to SRH services. This would equip deaf persons with the necessary information to be aware of their reproductive rights and the services available to them and assert their rights in the event of a violation.

## Data Availability

The datasets generated and/or analyzed during the current study are not publicly available due ethical restrictions but are available from the corresponding author on reasonable request.

## Abbreviations

ANOVA: Analysis of variance
GNAD: Ghana National Association of the Deaf
SRH: Sexual and Reproductive Health
